# Decoding Protein Corona Through Synchrotron-Based Small-Angle X‑Ray Scattering

**DOI:** 10.1021/acsomega.5c05541

**Published:** 2025-09-23

**Authors:** Juliana Tosta Theodoro Carvalho, Antônio Malfatti-Gasperini, Ben J. Boyd, Liming Wang, Mateus Borba Cardoso

**Affiliations:** † Brazilian Synchrotron Light Laboratory (LNLS), 215006 Brazilian Center for Research in Energy and Materials (CNPEM), 13083-100 Campinas, São Paulo, Brazil; ‡ Institute of Chemistry, University of Campinas (UNICAMP), CEP, 13083-970 Campinas, São Paulo, Brazil; § Drug Delivery, Disposition and Dynamics, Monash Institute of Pharmaceutical Sciences, 2541 Monash University, Parkville, VIC 3052, Australia; ∥ CAS Laboratory for Biomedical Effects of Nanomaterials and Nanosafety, Laboratory for Interface Science and Biology, Institute of High Energy Physics, Chinese Academy of Sciences, Beijing 100049, P. R. China

## Abstract

Nanoparticles (NPs) in biological environments rapidly become coated with a dynamic biomolecular layer known as the protein corona (PC), significantly influencing their biological identity and functionality. Traditional methods used to characterize the PC often disrupt its native state, limiting accurate insights into its true structural and compositional complexity. Synchrotron-based small-angle X-ray scattering (SAXS) provides a powerful alternative, enabling nondestructive, label-free, and in-solution analysis of the PC under physiologically relevant conditions. This minireview critically examines recent advancements in applying SAXS to decode the PC, highlighting methodological developments and exemplary studies demonstrating SAXS’s unique ability to resolve interactions at the nano–bio interface. By discussing novel analytical frameworks, such as integrating SAXS with complementary techniques like small-angle neutron scattering (SANS) and cryo-transmission electron microscopy (cryo-TEM), we provide a comprehensive overview on the structural and thermodynamic features of the PC. Furthermore, we outline future opportunities including time-resolved SAXS to elucidate the kinetics of corona formation and the establishment of standardized protocols to enhance reproducibility and reliability. Ultimately, this review positions SAXS as an indispensable tool for advancing our understanding of nanoparticle–protein interactions and fostering innovation in nanomedicine.

## Graphical abstract

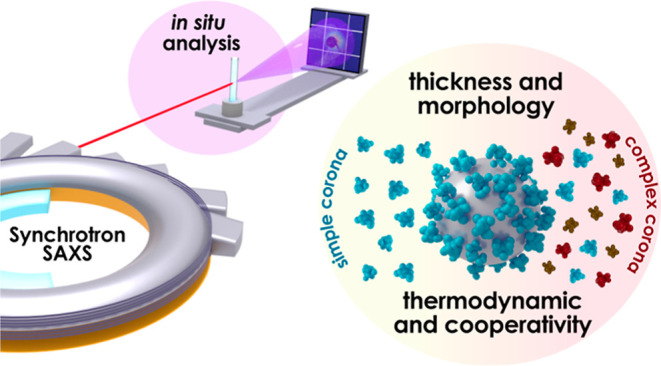

## Introduction

The interface between two entities, such as particles and physiological fluids, is the sweet spot where the origin of physicochemical phenomena leads to desirable or undesirable outcomes. More specifically, when referring to nanomaterials, this interface is more reactive and consequently more prone to interact with the external environment than bulk materials.^1^ As a result, the favorable properties of nanomaterials have encouraged widespread research into their performance and efficiency in various fields. Nanobio phenomena are a class of events essential to understanding the interactions of nanomaterials with, for example, tissues, cells, biomolecules, and biological fluids.^2^ These complex relationships between nanomaterials and components of the human body give rise to an entire field of study devoted to revealing nanobiological events. Understanding these relationships more deeply was the impetus for a seminal work in 2007 in which Kenneth Dawson and collaborators first coined the term ‘protein corona’ (PC),^3^ to describe the phenomenon of proteins adsorbing to the surface of nanoparticles (NP) in physiological fluids (Figure 1a). Since then, numerous works have emerged with experimental evidence seeking to understand, explain, and predict the formation of the PC, its relation to NP attributes, and its subsequent biological consequences in the human body.^4^ The PC is generally classified into two layers: a hard corona, formed by proteins with high affinity and long residence times on the NP surface, and a soft corona, composed of weakly bound proteins that exchange rapidly with the surrounding environment.^5^ Although the formation of the PC remains the most extensively studied phenomenon in the field of bionano interactions, it is now widely recognized that NP surfaces can also be covered by a diverse range of biomolecules present in real biological environments. Under physiological conditions, the corona is not exclusively composed of proteins but rather constitutes a dynamic and complex layer known as the biomolecular corona. Nevertheless, in intravenous environments, where proteins are the most abundant macromolecules in plasma, the corona is predominantly protein-based. This predominance explains the extensive focus of the community on the PC over the past two decades. Accordingly, this review primarily focuses on the PC, while also acknowledging the emerging relevance of the biomolecular corona concept, which is increasingly being recognized as a critical factor for understanding NP behavior in complex biological systems. Several studies have explored the qualitative and/or quantitative characterization of the composition, structure, and dynamics of the PC, in addition to proteomics-based techniques.^6–10^ Various methodologies and techniques are dedicated to these studies in the literature, based on spectroscopy,^7^ fluorescence,^11^ transmission electron microscopy,^5^ and scattering (dynamic light scattering and small-angle X-ray scattering).^6,12,13^ Of these techniques, scattering techniques are possibly the least well understood by those working in the PC field, hence increasing this familiarity is one objective of this review article.

**Figure 1:**
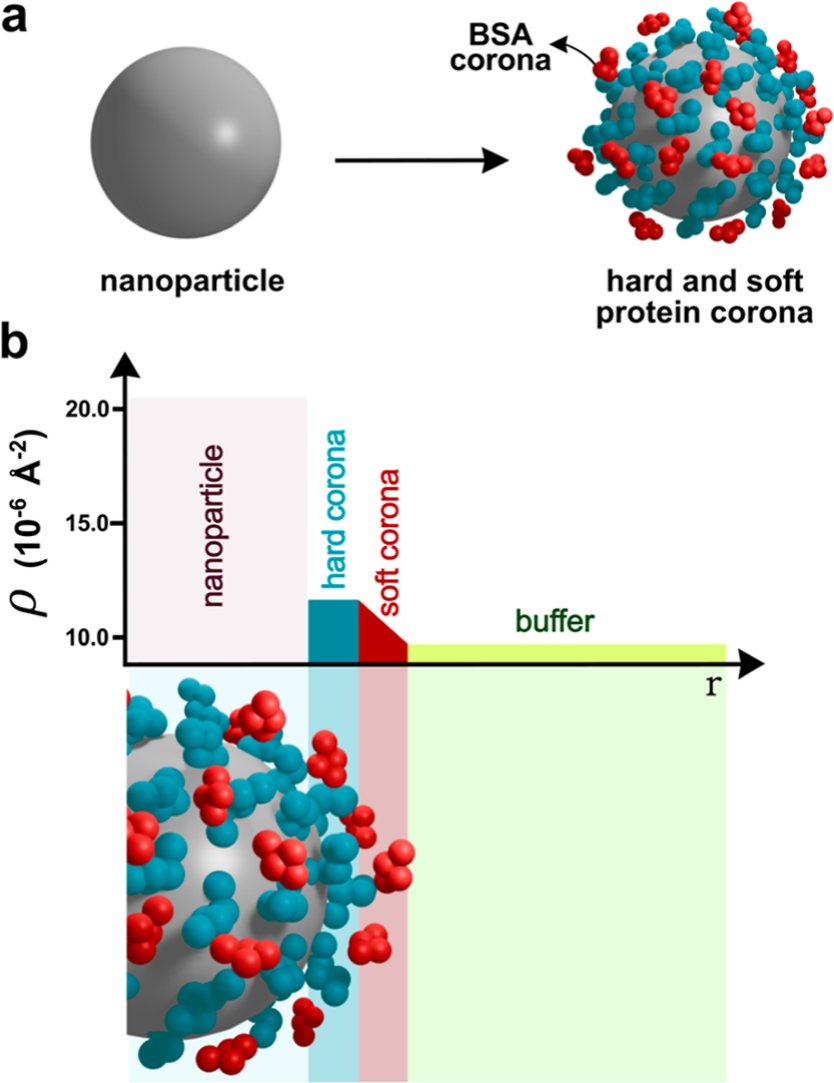
(a) Schematic representation of protein corona formation, including the hard (blue) and soft (red) corona. (b) Schematic representation of the electron density (ρ) profile across a nanoparticle–protein corona system in a buffer environment as a function of radial distance (r). Electron densities correspond to silica particles, bovine serum albumin and phosphate buffer.

Shedding light on the unknown is a scientific practice that enables discoveries and continually pushes the frontiers of knowledge. Particularly, the intentional interaction of light with matter can yield results that answer relevant social and scientific questions. In this scenario, X-rays can reveal details and allow indirect visualization of the organization, structure, and composition of a wide range of materials. Critically, X-rays are in the range of wavelengths where they can interact with matter at nanometer length scales leading to either absorption by or scattering from the material.^12,14^ X-rays provide detailed structural information about a specimen through scattering, where the structure arises from differences in electron density due to the packing of molecules in a material or from interfaces between material and solvent. Small-angle X-ray scattering (SAXS) aligns with this premise and is an extremely efficient and widely employed characterization technique for structural analysis. The PC is a dynamic mono or multilayer of proteins, typically in the range of a few nm in thickness, whose composition and structure evolve over time.^15^ However, analyzing the PC using SAXS presents a few challenges described in this review, due to the low difference in electron density between the (hard and soft) PC and the surrounding medium, mainly water or buffer solutions. Figure 1b provides a conceptual representation of the hard and soft PC in the context of SAXS analysis. The model BSA hard corona (blue) is characterized by a quasi-uniform electron density, consistent with a densely packed and structurally stable protein layer tightly bound to the nanoparticle surface. In contrast, we suggest that the BSA soft corona (red) can be described by a smoothly decaying electron density profile, that diminishes progressively until it becomes indistinguishable from the surrounding buffer (green). In addition, the mismatch in size between the NP and the thickness of the PC makes it particularly challenging to determine the detailed attributes of the PC.

This review explores how SAXS can be used to investigate the complex interactions between biological entities, such as proteins, and nanoparticle surfaces within the context of nanomedicine. We begin by outlining the fundamental principles of SAXS, highlighting its unique capabilities in probing nanoscale structures in solution. We then discuss its potential for characterizing PC formation, emphasizing its ability to provide qualitative, quantitative, and thermodynamic insights into these dynamic interfacial phenomena, as well as highlighting various limitations. Further, we present case studies where SAXS has successfully unveiled critical aspects of PC on nanoparticles of diverse compositions, reinforcing its role as an essential technique for advancing our understanding of the PC phenomena. Finally, we present practical strategies for planning synchrotron-based SAXS experiments, highlighting key aspects ranging from sample preparation and beamline selection to data acquisition parameters, with an emphasis on actionable recommendations derived from hands-on experience.

## Small-Angle X-Ray Scattering: Principles and Methodology

Nanomaterials can be effectively evaluated using SAXS, allowing for the observation of their intrinsic properties in their native state or in physiological fluids.^16^ This feature holds great significance in research on soft matter since nanomedicines are intended to function in sophisticated settings such as human biological frameworks. SAXS analyzes the entire population of particles in solution, providing ensemble-averaged structural information that is statistically representative, unlike techniques such as transmission electron microscopy, which examine only a limited number of isolated particles. Samples for this approach are usually in either liquid or solid states. In synchrotron-SAXS experiments, a monochromatic and well-collimated X-ray beam passes through the sample, which is positioned in its path. The beam is scattered by regions with different electron densities within the sample, generating a scattering pattern (Figure 2a). This configuration corresponds to SAXS in transmission mode, in which the scattered signal arises from X-rays that go across the full thickness of the sample. A given image is then captured by a 2D detector as a scattering pattern and reflects the overall structural organization of the sample. The final data corresponds to the average scattering signal from the illuminated area. This 2D pattern is then subjected to an azimuthal integration, generating a 1D scattering profile (Figure 2b). This step assumes the system is isotropic, meaning the structural features are randomly oriented, resulting in uniform scattering intensity along all azimuthal angles. Indeed, the name of the technique derives from analyzing the scattering pattern at small angles, usually in the range of 0.1° to 5°. During a SAXS measurement, the intensity of X-ray scattering (*I*) is obtained as a function of the scattering vector (*q*), which depends on the scattering angle of the X-rays relative to the incident beam (2θ) and the X-ray wavelength.^14^ The scattering vector q is determined as *q* = 4 π/λ sin θ, where λ is the wavelength of the incident X-ray, usually defined in nm^–1^ or Å^–1^, depending on the unit used for λ. The unique interaction of the sample’s constituents with X-rays results in a distinct scattering pattern. Additionally, the scattering pattern of materials made of heavier atomic number elements, i.e., those with more electrons and hence a greater electron density, will be more prominent. This attribute is directly related to the chemical composition of the sample. A limitation worth discussing in SAXS analysis of the PC is the significant contrast difference between the NP, the most prominent scattering object in the system, and the thin layer of biomolecules forming the corona. Furthermore, the electron density of the PC is often similar to that of the surrounding medium (e.g., buffer solutions), making its discrimination considerably challenging. Synchrotron-based SAXS has helped lessen this issue, as the high brilliance of the X-ray beam enhances the sensitivity to subtle differences in scattering. This enables better distinction of the corona’s contribution, even when the contrast between the PC and the surrounding medium is low.

**Figure 2:**
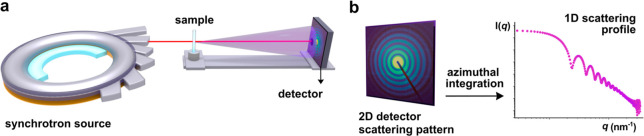
(a) Small-angle X-ray scattering (SAXS) setup at a synchrotron source. The X-ray beam, generated by the synchrotron, interacts with the sample, and the scattered intensity is recorded by a detector, enabling structural characterization of nanoparticles and their protein corona in solution. (b) The 2D SAXS scattering pattern collected by the detector undergoes azimuthal integration to generate the corresponding 1D scattering profile, *I*(*q*) versus *q* in log–log scale.

The scattering signal in SAXS results from electron density differences within the particle and relative to the solvent, allowing structural features to be resolved. The more significant the difference between the sample and its environment regarding electron contrast, the more prominent the signal becomes.^17^ Since each entity in a system has its own electron density (ρ), scattering occurs only when there is a nonzero contrast between the electron densities of the components, such as nanoparticles and the surrounding medium. In SAXS, scattering is dominated by size, meaning that larger components can readily overshadow the contribution of smaller ones. This primarily occurs because *I* increases with the square of the particle’s volume—that is, with the sixth power of the particle size. Depending on the specific parameters being investigated within the system, this property may represent either an advantage or a disadvantage. Moreover, SAXS obtains a statistical average of the shapes and positions of the sample with ease, robustness, and precision, which underpins the main advantage of using the technique: representativeness. Despite its low resolution, this technique offers advantages over complementary methods (such as imaging) since it delivers representative and highly accurate access to structural information across various sizes. Two key concepts related to the SAXS technique are the form (F­(*q*)) and the structure (S­(*q*)) factors. Since most particles in a given system (e.g., spherical NPs, cubosomes, rod-like NPs) are homogeneous in size and shape, the scattering signal will oscillate in a characteristic manner consistent with their physical shape. This characterizes the form factor in SAXS and generates a form-dependent interference pattern. Through form factor analysis, it is possible to extract information on the size, shape, and size distribution of the sample, primarily by interpreting or modeling the obtained scattering pattern.^17^

Likewise, the structure factor refers to the spatial arrangement of particles. It involves the presence of correlations between interparticle positions, meaning that the distances among all particles are repeated and maintained. This enables the determination of the relative positions of neighboring particles. Notably, SAXS allows the observation of the system on different dimensional scales, where it is possible to see evidence of the current state of NPs in the system. Distinct features along the scattering vectors (*q*) of a single SAXS curve correspond to distinct system properties.

Different types of X-ray sources are available for SAXS experiments, with synchrotron SAXS emerging as a particularly valuable opportunity to interrogate colloidal systems. When SAXS is performed using a synchrotron radiation source, the benefits in terms of photon flux, time resolution, and data quality are substantial. The high brilliance of synchrotrons, especially fourth-generation sources, results in lower beam divergence, which reduces background contribution from possible parasite scattering (i.e., scattering not provided from the sample) and consequently improves the distinction of sample features in SAXS experiments. Consequently, data integration is improved, providing high angular resolution and minimizing deviations in the scattering vector *q* (Δ*q*), thereby enhancing the reliability of the data.^18^ However, the increased photon flux also entails certain drawbacks, such as the need for greater caution regarding radiation damage and, consequently, potential harm to the sample, particularly considering the delicate nature of the PC. The type of SAXS experiment conducted on a synchrotron source depends on the configuration of the beamline and available sample environments. Currently, several beamlines are dedicated to synchrotron SAXS, enabling various experimental designs according to the features of each beamline.

Most of the synchrotron-based SAXS beamlines work in transmission mode where a minimal sample volume is typically required (usually <100 μL), allowing for sample recovery due to the nondestructive setups available in most of the beamlines.^19,20^ Liquid sample analyses are commonly conducted in thin X-ray inert capillaries, typically about 1 mm thick, where the sample moves in front of the beam to avoid specimen degradation. The analysis of samples containing low-contrast features, such as a PC, requires careful background subtraction in order to isolate the scattering signal of interest accurately. Measurements of the sample and buffer for subtraction must be taken at the same capillary position, primarily because the capillary wall itself is heterogeneous, which can introduce unwanted scattering contributions in the scattering pattern and make careful background subtraction impossible. Furthermore, the quality of the data obtained is directly related to the carefulness of the experimental design. Robust, reproducible, and comparable data are the natural consequence of good sample preparation. An alternative sample environment for a satisfactory buffer subtraction comprises a sealed cell of two thin, parallel mica windows. In this configuration, the sample can be either injected into the chamber or preloaded into the sample space before sealing.^21^ Thin mica windows can minimize the overall background of the SAXS camera and suppress undesirable and parasitic scattering contributions. Moreover, mica provides a smooth and homogeneous scattering profile in the SAXS region, which can be effectively subtracted, ensuring minimal interference with the sample signal.

For beamlines configured for high-throughput SAXS (HT-SAXS), i.e. systems designed to process a large number of samples in a short period, automated platforms incorporating 96-well plates and robotic arms are frequently employed.^20,22^ These setups enable automated sample injection, measurement, and cleaning, ensuring large sample sets. Additionally, prior knowledge of the background components and their possible influence on the scattering profile is pivotal, given that every element in the sample will generate a desirable or undesirable scattering profile. This is especially critical for background subtraction, where the ideal scenario is to obtain a background (e.g., buffer) measurement before measuring the sample. In some cases, it is advisible be aware of any potential artifacts that might compromise the buffer or background subtraction process, thereby hampering the final integrated curve. Selecting the appropriate pair of sample and buffer curves for subtraction and integration is essential. SAXS is often combined with other structural characterization techniques to better characterize the nature of a system from different perspectives. Two techniques that can be effectively combined with SAXS are small-angle neutron scattering (SANS) and cryo-transmission electron microscopy (cryo-TEM). Table 1 concisely compares SAXS, SANS, and cryo-TEM, highlighting their key advantages and limitations. This comparative overview is intended to offer a strategic framework from which one can determine how to best combine these techniques, depending on the scientific case regarding PC analysis.

**Table 1:** Comparison of Synchrotron SAXS, SANS, and Cryo-TEM Regarding Sample State, Information Obtained, Main Advantages, and Limitations^a^

	**SAXS**	**SANS**	**cryo-TEM**
type of radiation	*X-rays*	*neutrons*	*electrons*
sample state	solution	solution	vitrified (frozen) grids
information obtained	low-resolution morphology, size distribution, aggregation state, thermodynamic parameters	same as SAXS	high-resolution morphology, atomic or near-atomic structure
main advantages	representiveness, label-free, nondestructive, fast data acquisition, native-like conditions	no radiation damage, contrast matching, suitable for complex soft matter	direct visualization of individual coated particles
limitations	low resolution compared to microscopy, limited for heterogeneous or highly complex systems	slow data acquisition, low flux, high sample volume required	sample preparation artifacts (e.g., freezing artifacts), low throughput, potential bias in particle selection

a The table was elaborated based on previous experience and expertise of the authors and cross-validated with literature data.

The following section presents case studies employing SAXS to elucidate various aspects of the protein corona, some of which demonstrate the enhanced value obtained through its combination with SANS and/or cryo-TEM.

## Expanding SAXS Applications to Protein Corona Characterization

SAXS is a mature and well-established technique, but it has only intersected with studies focused on elucidating the PC in recent years.^9^ PC composition varies depending on the surrounding matrix and is strongly influenced by physicochemical properties such as particle size and surface chemistry.^23–25^ Each unique biological context gives rise to a corona with specific characteristics governed by the availability of biomolecules in the environment and the nanoparticle’s surface properties.^26^ Predicting and controlling these interactions is critical in nanomedicine and remains one of the significant challenges, particularly due to the high degree of heterogeneity and the dynamic nature of the biological milieu. Given the complexity and context-dependent behavior of the PC, it is essential that experimental studies be conducted under conditions that closely mimic physiological environments. To address this unpredictability, advanced synchrotron-based techniques, such as SAXS, offer powerful tools for designing and implementing more realistic and robust experimental approaches.

Indeed, it is known that the presence of the PC and its composition are directly responsible for the macroscopic behavior of particles in each medium, potentially leading to phenomena such as aggregation.^4^ Regarding the analysis and interpretation of the data obtained, a fundamental and mature understanding has not yet been reached to study specific surface interactions between NPs and biomolecules, which give rise to the PC. Satisfactorily, it is possible to characterize one or the other individually but analyzing the scattering profile after the corona formation still needs extra care. Most studies on the PC rely on separating NPs from the surrounding medium, a process that often disrupts the loosely bound proteins of the soft corona. In contrast, SAXS allows for *in situ* analysis without the need for separation, preserving the native state of both the hard and soft corona. Nevertheless, this represents only a preliminary exploration of the field, and dedicated works that propose new adjustments and bolder data analyses are still emerging.

Spinozzi and colleagues^9^ proposed a comprehensive approach to analyzing human serum albumin (HSA) structure and thermodynamics associated with gold NPs combining SAXS and SANS. This work focused on the PC’s stoichiometry, cooperativity, and structural properties. The dual approach leverages the complementary contrast mechanisms of SAXS and SANS to describe the binding process comprehensively. By varying the concentration of protein and NPs, they collected a sequence of scattering patterns and analyzed the entire data set, revealing that the PC process follows the Hill dissociation model. The thermodynamics, characterized by the standard Gibbs free energy change (Δ*G*°) and the Hill model’s *n* parameter, indicated the formation of a soft corona around the NPs, which decreased the affinity for additional protein molecules. The NP-HSA structure was modeled as a three-density-level sphere: an inner core of gold NPs, an intermediate layer of capping citrate anions, and an outer monolayer of HSA (Figure 3a).

**Figure 3:**
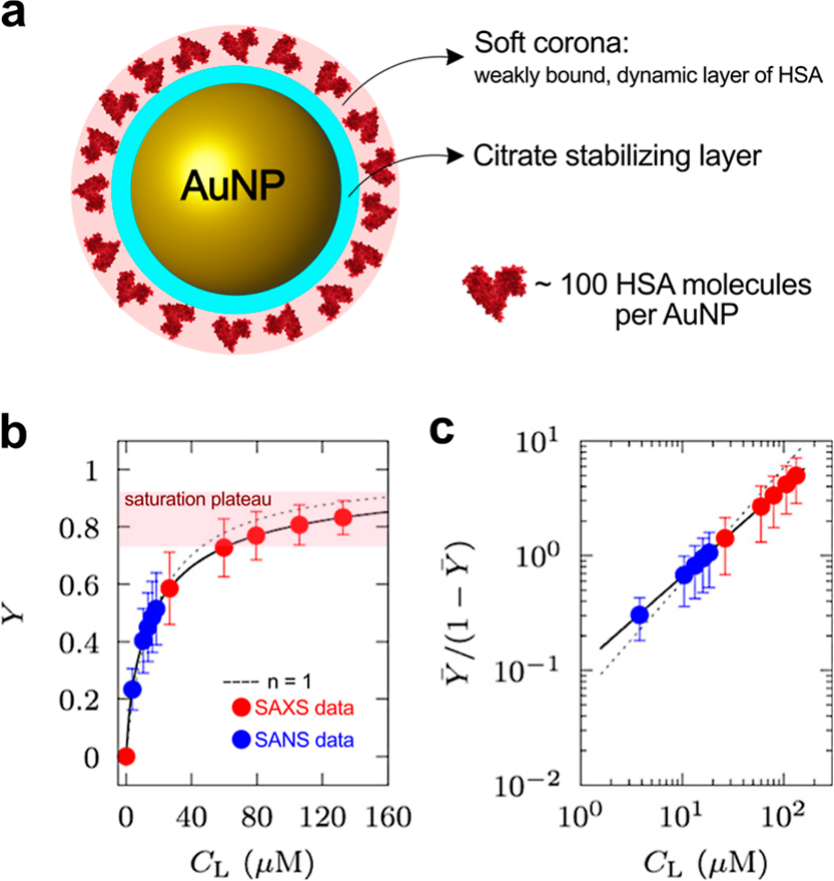
(a) Schematic representation of the protein corona formed around AuNPs. The nanoparticles are initially stabilized by a citrate layer, and a soft corona of weakly bound, dynamic HSA molecules form upon interaction. (b) Binding isotherm of human serum albumin (HSA) on gold nanoparticles (AuNPs) obtained from SAXS (red) and SANS (blue) data. The fraction of bound HSA (*Y*) is plotted as a function of the total HSA concentration (*C*_L_), showing a saturation plateau at high concentrations. (c) The same data in (b) as a Hill plot representation. Panel (b,c) reproduced after modification from ref 9, Copyright © 2017, American Chemical Society.

Figure 3b shows the SAXS (red) and SANS (blue) data sets, fitted using a binding model, which indicates a maximum number of ∼100 HSA molecules per nanoparticle regardless of the technique used, confirming the model’s reliability. The Hill plot analysis (Figure 3c) further demonstrates that the adsorption follows negative cooperativity, meaning that as more proteins bind, subsequent binding becomes progressively less favorable. This behavior is attributed to steric hindrance and electrostatic repulsion among adsorbed proteins, forming a soft, dynamic protein corona rather than a densely packed monolayer. Moreover, data were collected under physiological conditions, highlighting the technique’s potential for *in situ* (within the native environment) and *in operando* (under active, functional conditions) studies of nanoparticle–protein interactions.

More recently, Ferreira and colleagues^13^ employed SAXS to distinguish between the formation of PC and the aggregation phenomenon in model silica NPs while exposed to various protein sources and media with different ionic strengths. Figure 4a (top panel) presents selected SAXS curves obtained from mixtures of SiO_2_ and lysozyme in phosphate buffer (PB) for distinct protein-to-particle molar ratios (PPMR). Notable variations in the scattering profiles reflect changes in the sample composition, particularly due to aggregation phenomena or shifting proportions between components. In the low-*q* region, the signal is primarily influenced by distinct structural entities (either individual nanoparticles at lower PPMRs or free proteins at higher PPMRs as well as by the emergence of aggregates), which significantly alter the slope of the scattering curve in this range. In contrast, for large *q*-values, the scattering is governed by the independent contributions of SiO_2_ particles, proteins, or their simple physical combination, as no evidence of cooperative interaction was observed. The authors then applied a deconvolution approach incorporating up to three distinct scattering components: (a) SiO_2_ nanoparticles modeled as polydisperse spheres, (b) proteins, and (c) aggregates, the latter described by a power-law decay with a cutoff radius constrained by the characteristic SiO_2_ particle size (Figure 4a bottom). For all fits, the scattering of pure SiO_2_ and protein solutions served as fixed templates, with only scaling factors adjusted in the mixtures, significantly minimizing the number of adjustable parameters.

**Figure 4:**
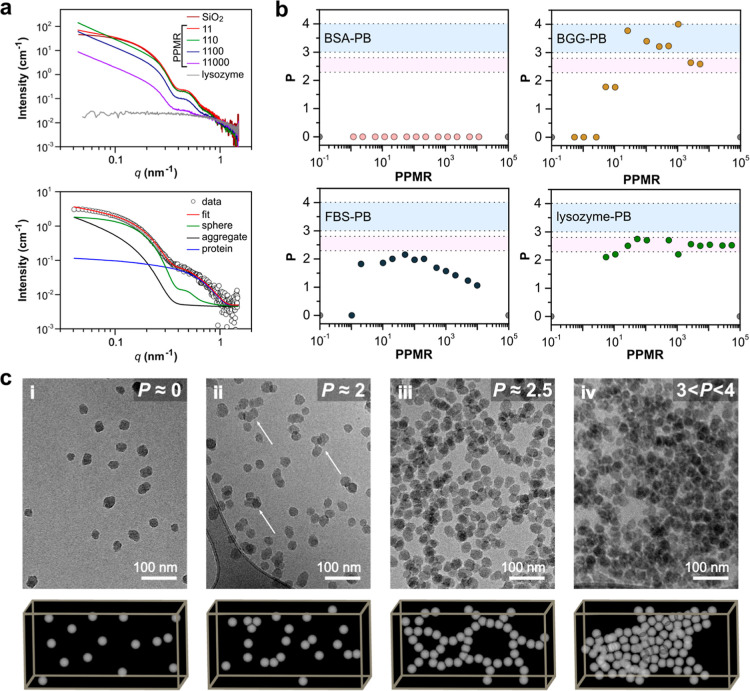
(a) SAXS profiles of pure lysozyme, pure SiO_2_, and selected mixtures at different protein-to-particle molar ratios (PPMR) in phosphate buffer (PB), and SAXS curve of a BSA-SiO_2_ sample at PPMR = 20, highlighting structural contributions used in data fitting: global fit (red), BSA signal (blue), SiO_2_ signal (green), and aggregate contribution (black). (b) Power-law decay (P) evolution as a function of PPMR for BSA, BGG, lysozyme, and FBS in PB buffer. (c) Cryo-TEM images and schematic representations of SiO_2_ interacting with different proteins at varying PPMR. Samples include: (i) BSA (*P* = 0), (ii) FBS (*P* = 2.0), (iii) lysozyme (*P* = 2.5), and (iv) BGG (3 < *P* < 4). Adapted with permission from ref 13, Copyright © 2022, American Chemical Society.

By integrating SAXS analysis, specifically the power-law decay behavior in the low-*q* region, with cryo-TEM, it was possible to distinguish between simple PC formation, the presence of small aggregates such as dimers or trimers, and the presence of large aggregates. In SAXS, the power-law decay describes how the scattering intensity *I*(*q*) varies with the scattering vector *q*, following the relationship *I*(*q*) ∝ *q* ^–*P*^. The exponent *P* provides key structural insights into the system. Values of *P* range from 0 to 4: *P* = 0 indicates homogeneous entities with no aggregation and, consequently, corona-free systems; *P* ∼ 2 suggests the formation of small, low-density aggregates; *P* ∼ 2.5 corresponds to mass fractals; and *P* > 3 indicates surface fractals, associated with dense aggregate structures.^13,27^ Figure 4b illustrates this phenomenon, where the Job’s plots (*P* × PPMR) highlight distinct structural regimes, where the highlighted pink region corresponds to mass fractals, while the blue region indicates surface fractal behavior. These plots correlate with the cryo-TEM images (Figure 4c), which visually demonstrate the evolution of the PC as a function of *P*, ranging from a simple, homogeneous corona (*P* ≈ 0) to increasingly complex and massive aggregate structures (3 < *P* < 4), depending on the biological medium and ionic strength.

A similar SAXS approach was recently employed to cross-validate results obtained from X-ray photon correlation spectroscopy (XPCS), a novel and highly promising synchrotron-based technique for probing the dynamics of nanoparticles and proteins in complex environments.^10^ In this framework, SAXS data played a critical role in differentiating the dynamic behavior of bare and functionalized silica NPs across varying levels of protein complexity, thereby reinforcing the robustness and reliability of the findings. When combined with XPCS-derived dynamic information, SAXS enabled the decoupling of static protein adsorption effects from collective diffusive behavior. Alternatively, Galdino and colleagues^5^ introduced a new method for characterizing a BSA or FBS corona on silica NPs, accurately estimating binding parameters. By monitoring a known amount of protein added to interact with silica NPs, they observed a vertical curve shift in the low-*q* region of SAXS scattering curves (Figure 5a). Furthermore, the authors proposed a novel SAXS data analysis strategy where Kratky plots (*I* × *q* *^2^* vs *q*) with classical bell-shaped curves resembling spherical entities were obtained. The Kratky maximum peak can shift in intensity and position, indicating changes in the system (Figure 5b). By analyzing both SAXS curves and Kratky plots, the authors found evidence of the formation of the PC. Specifically, the Kratky maxima increased and moved to lower *q* values, which indicates a size increase due to the protein adsorption. Additionally, a binding isotherm curve was derived by fitting the Kratky maxima values to a Hill isotherm (Figure 5c), revealing positively cooperative behavior with a Hill coefficient of *n* = 1.6 (*n* > 1).

**Figure 5:**
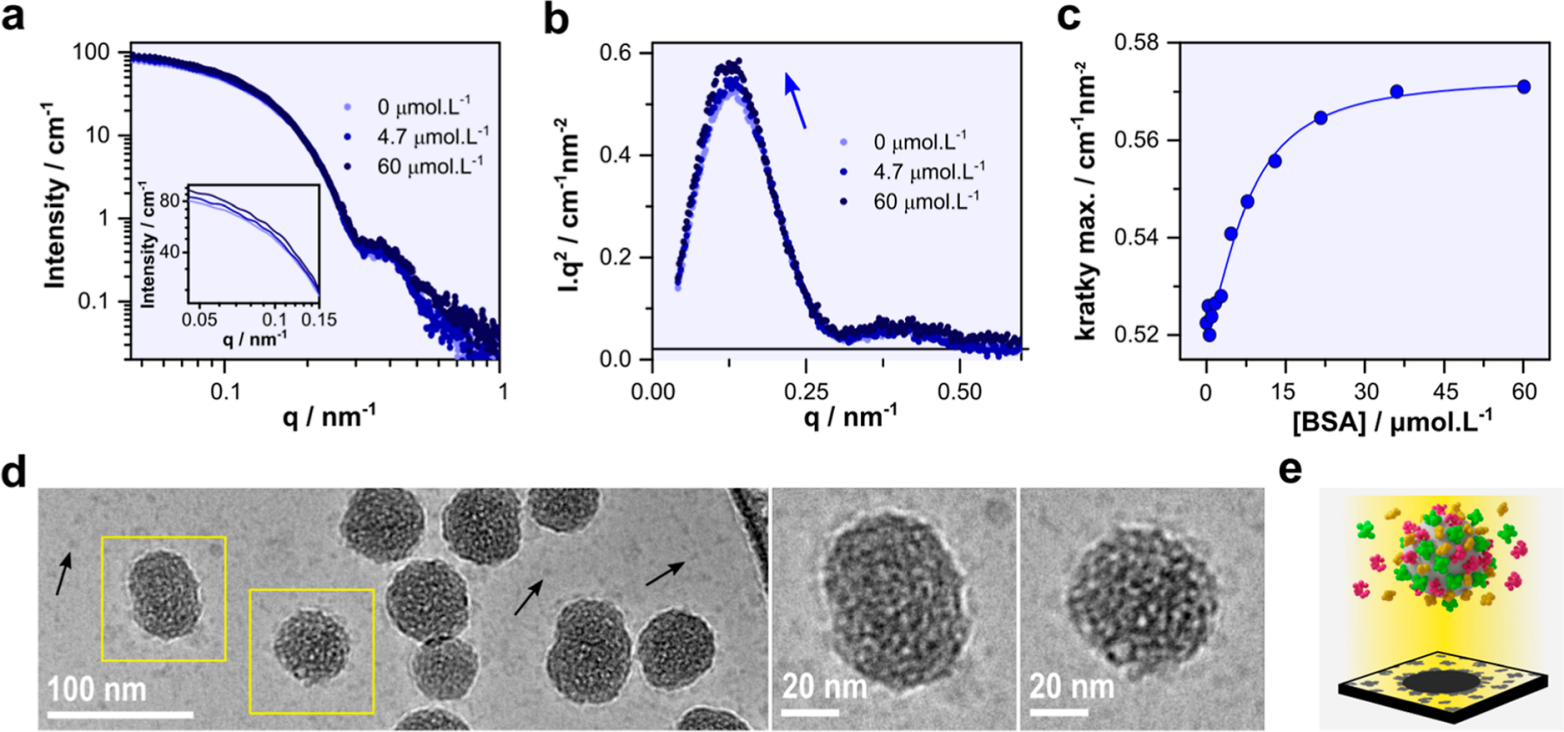
(a) SAXS curves in log–log scale, (b) Kratky plots, and (c) Kratky plot maxima as a function of BSA concentration, demonstrating protein corona formation on SiO_2_. (d) Cryo-TEM micrograph of 0.1 g·L^–1^ SiO_2_ in RPMI with 10% FBS after cross-correlation image processing and summation and expanded views of the nanoparticles, as indicated by the yellow boxes. (e) Schematic illustration representing the protein corona in a multiprotein system. Reprinted with permission from ref 5, Copyright © 2021, American Chemical Society.

In addition to SAXS data, the authors also investigated fetal bovine serum (FBS) corona formation using cryo-TEM with cross-correlation and summation techniques. While traditional cryo-TEM imaging failed to resolve individual proteins due to their low contrast, cross-correlation image processing and summation of multiple micrographs significantly enhanced the visibility of both free and adsorbed proteins on nanoparticles (Figure 5d). This approach confirmed the presence of a heterogeneous corona composed of varying-sized proteins (5–8 nm). The schematic representation in Figure 5e illustrates the structural complexity of the multiprotein corona, reinforcing the need to corroborate SAXS-based structural findings with direct imaging techniques. This is a successful example in which scattering and imaging techniques complement each other in describing the wholeness of a PC system.

Looking at a very complex corona approach, a specific variation of the corona relevant to orally administered nanomedicines, known as the gut corona or gastrointestinal (GI) corona, was recently elucidated using SAXS.^28^ The authors revealed the structure of the gut corona across different levels of intestinal fluid complexity, ranging from simplified model systems (isolated bile salts) to biologically relevant fluids (extracted bovine bile) (Figure 6a). Additionally, the composition of the hard corona was analyzed using liquid chromatography with tandem mass spectrometry (LC–MS/MS). As emphasized by the authors, bile is a complex biological fluid that plays a crucial role in the interaction of NPs within the GI environment. It consists of bile salts, phospholipids, proteins, and other biomolecules. The key distinction of this study is its focus on interactions between NPs and nonprotein biological components, which can modulate NP behavior as significantly as the PC, an aspect often overlooked in conventional studies. The authors successfully applied the raspberry model to fit the SAXS experimental data, providing insights into the adsorption behavior of simulated bile fluids with increased complexity onto silica NPs. The sticky hard sphere model was employed to describe the attractive interactions between the assembled raspberry-like particles.

**Figure 6:**
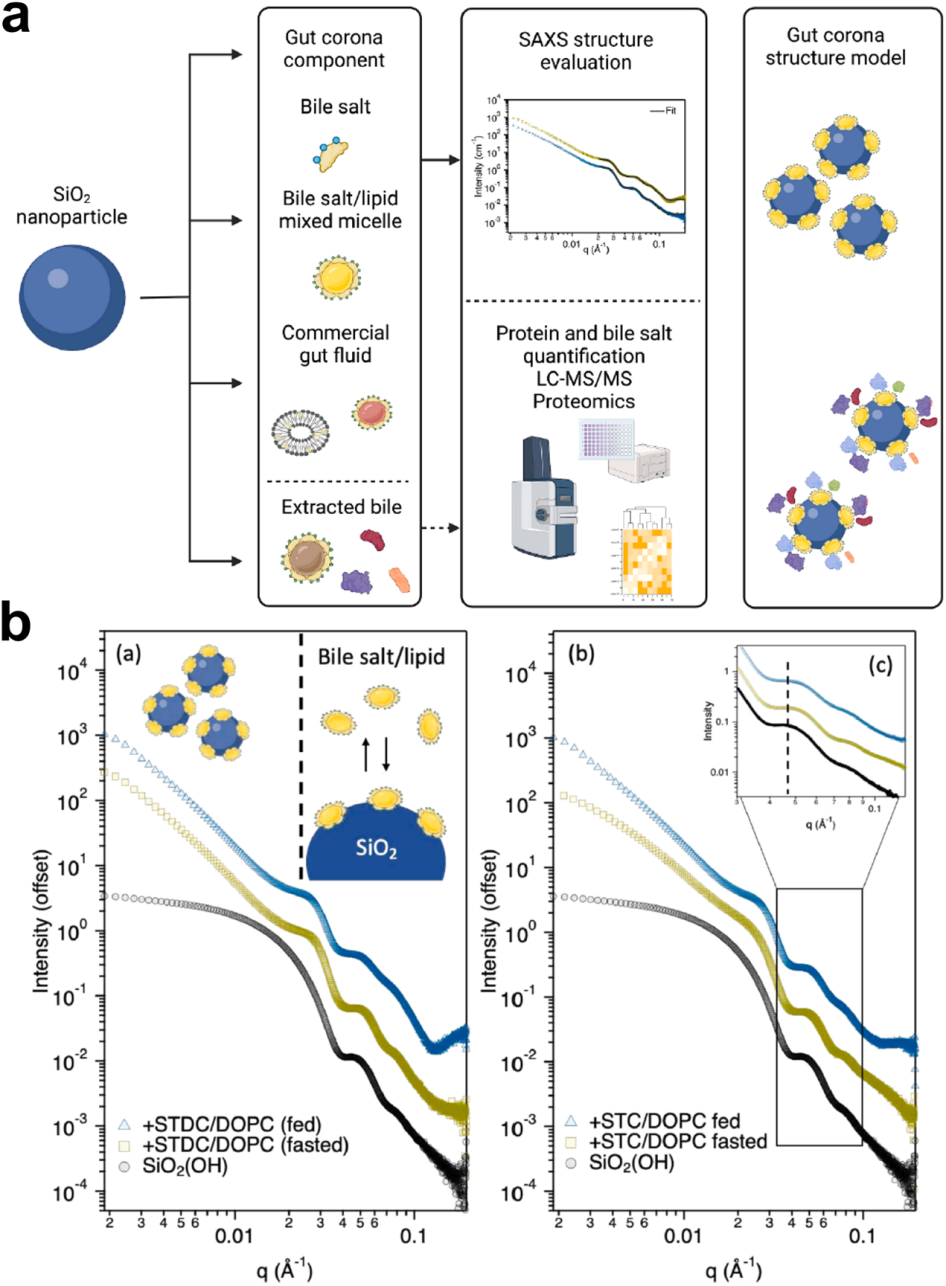
(a) Schematic workflow illustrating gut corona formation in simulated bile fluids, with structural analysis performed by SAXS and corona composition analysis conducted with LC–MS/MS. (b) SAXS curves of silica NPs in the absence and the presence of STDC/DOPC and STC/DOPC mixed micelles. The magnified region of the graph highlights a shift in *q* when mixed micelles are present, under fed and fasted conditions. Reprinted from ref 28, © 2024 The Author(s), Published by Elsevier Inc., with permission.

In this context, the raspberry model outperformed commonly used approaches such as sphere, core–shell and sticky hard sphere models to describe the corona formation. This model suggests that the NP + bile components form a heterogeneous corona, where the micelles do not distribute as a uniform layer but rather as clusters, resembling a raspberry-like structure. For instance, when mixed micelles composed of sodium taurodeoxycholate (STDC) and 1,2-dioleoyl-*sn*-glycero-3-phosphocholine (DOPC) adsorbed onto silica nanoparticles (NPs), a shift in the scattering vector was observed at *q* = 0.045 Å^–1^, moving toward a slightly higher *q* value. This shift strongly indicates the effective adsorption of the micelles onto the silica surface (Figure 6b). The authors further discussed the role of surface functionalization in the colloidal stability of the NPs under the studied conditions. While NH_2_-functionalized silica NPs remained stable, nonfunctionalized silica NPs showed aggregation. This work represents a compelling case where a heterogeneous and complex corona is formed, deviating from the classical monolayer protein adsorption model typically observed in model systems, such as single-protein studies. Ultimately, this study reinforces the capability of SAXS to characterize both model systems and biologically relevant complex coronas, advancing the understanding of nanoparticle behavior even under physiologically relevant conditions.

As demonstrated by the previous examples, SAXS can serve as a pivotal technique for evaluating various aspects of the PC, including its thickness, protein-to-NP ratio, morphology, and even thermodynamic properties. Its capabilities are significantly enhanced when combined with complementary techniques, notably SANS and cryo-TEM. By integrating SAXS with such methods, one can achieve a more comprehensive and robust characterization of the PC, thereby reinforcing the depth of the findings.

## Planning a Successful Nanoparticle–Protein Corona SAXS Experiment

Performing experiments at a synchrotron facility often requires the significant mobilization of a research team, substantial investment of time, and physical effort, as beamtime typically involves long hours of continuous measurements. The experimental planning phase is arguably the most critical. Unexpected challenges frequently accompany beamtime, and better-prepared users are more likely to use the beamtime more efficiently, thereby increasing the chances of a successful experiment. Below, we outline a few considerations for planning a successful SAXS experiment focused on the characterization of the PC on NPs. These suggestions focus on users with little or no prior experience with the technique. First, the *q*-range selected for a SAXS experiment must be carefully considered according to the characteristic sizes of the sample components (e.g., size of the NP). To ensure that the expected structures are properly resolved, it is crucial to determine both the minimum and maximum *q*-values, thereby covering all relevant structural information. This aspect is particularly critical for small nanoparticles, as demonstrated in an interlaboratory study by Pauw et al.,^29^ which showed that even small uncertainties in *q* calibration can lead to substantial errors in derived parameters. The lower accessible *q*-range in the experiment defines the largest structural feature that can be studied. Before applying for SAXS beamtime, verifying the *q*-range of the desired beamline is essential, ensuring that the sample features fall within the accessible range. Once it is confirmed that the NPs and PC structures fit within the beamline’s *q*-range, the correct experimental setup must be chosen. Complementary scattering techniques are available at synchrotron facilities that extend the capabilities of SAXS and enhance structural characterization across different length and time scales. Wide-Angle X-ray Scattering (WAXS) is a distinct technique from SAXS that probes smaller structures at higher scattering angles. Ultra-Small-Angle X-ray Scattering (USAXS), on the other hand, is particularly valuable for studying larger structures, making it especially suitable for NP-PC systems due to its ability to access even lower *q*-ranges. Time-resolved SAXS (TR-SAXS) techniques can provide insights into the temporal evolution of nanoscale configurations and PC organization. While stopped-flow TR-SAXS is commonly used to monitor fast mixing events, other approaches employing various triggering mechanisms (e.g., temperature, chemical jump) and sample environments have broadened the accessible time scales. TR-SAXS methods are particularly relevant for capturing the kinetics of PC formation and assembly on NP, as they can track complex assembly dynamics in real-time.^30,31^ Ensuring proper sample preparation, controlling experimental conditions, and systematically verifying data quality as it is acquired are key aspects of obtaining high-quality and reproducible SAXS measurements, ensuring a successful experiment. Proper sample preparation plays a fundamental role in obtaining high-quality SAXS data, as sample homogeneity and stability directly affect the robustness of the measurements.With the continuous advancement of novel *in-line* technologies, new opportunities arise for experimental design to achieve reliable and robust results. Recent developments have demonstrated that coupling Asymmetric Flow Field-Flow Fractionation (AF4) with SAXS enables size-based fractionation of polydisperse samples before scattering analysis.^35^ This approach allows the isolation of more homogeneous sample fractions, such as colloidally stable particles separated from aggregates. Another critical factor to consider is radiation-induced damage to the sample, which is governed by the total absorbed dose (a function of both exposure time and beam intensity) and the sample’s intrinsic radiation sensitivity. These parameters should be optimized and discussed with the beamline scientist assisting during the experiment. Radiation dose can be tuned by adjusting exposure time, modulating beam intensity, modifying beam geometry, or using sample holders that allow sample movement, ensuring fresh sample exposure to the beam.^32,33^ Radiation damage can also be monitored by observing the scattering intensity at *I*(0), the low *q* region of the SAXS curve, which may indicate sample degradation. This is typically manifested as the appearance of a slope at the low *q* region of the scattering curve, reflecting the possible formation of larger scattering structures.^32^ An additional strategy to reduce radiation damage and avoid sample accumulation on capillary walls is using sheath-flow (coflow) systems, where the sample is confined to the center of the capillary by an outer buffer stream. This setup has enhanced dose tolerance and improved data quality in flow-based SAXS experiments.^34^ During SAXS measurements, capillary cleanliness must be continuously monitored in this setup. Proteins and NPs may attach to the capillary walls, leading to artifacts in subsequent measurements. A standard quality control step is to measure the buffer alone between sets of sample acquisitions. If residual scattering from the sample is detected (such as NP scattering profile, even at very low intensity), further cleaning is required.^35^ More recently, integrating synchrotron-based SAXS with Low-Frequency Raman (LFR) spectroscopy has allowed for simultaneous monitoring of structural and chemical transformations during *in situ* pH titrations.^36^ These innovative strategies represent significant progress toward enhancing SAXS-based investigations of NP-PC systems, especially under physiologically relevant conditions.

## Future Outlook

Synchrotron SAXS is an emerging and so far effective tool for characterizing the PC formation on a diverse range of NPs, offering insights into its impact on colloidal stability, structural organization, and thermodynamic behavior. Its unique ability to probe the corona directly in solution, under fully hydrated and physiologically relevant conditions, without the need for labeling, drying, staining, or physically separating hard and soft corona layers, provides a significant advantage over conventional methods. This feature preserves the native structure of the corona, enabling more accurate and representative analyses. While SAXS is firmly established in other fields, such as structural biology, its application in nanomedicine remains, at this moment, relatively underexplored. Looking forward, it is clear that further progress will depend on advancing experimental strategies, including tailored sample environments, modeling, and precise data acquisition pipelines. The development of standardized protocols and robust analytical frameworks is crucial for the nanomedicine community to fully leverage SAXS in PC studies. Such advancements will help ensure better data interpretation and prevent the underutilization of the technique, mitigating the risk of misleading conclusions. A particularly promising avenue lies in the use of TR-SAXS to investigate the kinetics of PC formation. This capability is critical for understanding how coronas assemble during the earliest moments of NP exposure to biological fluids. Applying TR-SAXS could shed light on transient states, adsorption dynamics, and the transition from soft to hard corona. Additionally, a possible frontier for SAXS in this context is moving toward quantitative analysis of PC, where future methodologies could be designed to distinguish and quantify bound and unbound proteins in solution. Furthermore, while it is recognized that bench experiments cannot fully replicate the complexity of real biological environments, it is likewise clear that *in situ* coronas formed under biologically relevant conditions offer a practical and accessible alternative for preclinical development. This approach provides researchers with greater flexibility, operational convenience, and the ability to probe systems under controlled yet meaningful conditions. Finally, we strongly advocate for using synchrotron SAXS as one of the main techniques for probing PC formation on NPs, offering peerless representativeness and versatility.

## References

[1] Nel A. E., Mädler L., Velegol D., Xia T., Hoek E. M. V., Somasundaran P., Klaessig F., Castranova V., Thompson M. (2009). Understanding Biophysicochemical
Interactions at the
Nano-Bio Interface. Nat. Mater..

[2] Ren J., Andrikopoulos N., Velonia K., Tang H., Cai R., Ding F., Ke P. C., Chen C. (2022). Chemical and Biophysical
Signatures of the Protein Corona in Nanomedicine. J. Am. Chem. Soc..

[3] Cedervall T., Lynch I., Lindman S., Berggård T., Thulin E., Nilsson H., Dawson K. A., Linse S. (2007). Understanding
the Nanoparticle-Protein Corona Using Methods to Quantify Exchange
Rates and Affinities of Proteins for Nanoparticles. Proc. Natl. Acad. Sci. U.S.A..

[4] Mahmoudi M., Landry M. P., Moore A., Coreas R. (2023). The Protein Corona
from Nanomedicine to Environmental Science. Nat. Rev. Mater..

[5] Galdino F. E., Picco A. S., Capeletti L. B., Bettini J., Cardoso M. B. (2021). Inside
the Protein Corona: From Binding Parameters to Unstained Hard and
Soft Coronas Visualization. Nano Lett..

[6] Giulimondi F., Digiacomo L., Pozzi D., Palchetti S., Vulpis E., Capriotti A. L., Chiozzi R. Z., Laganà A., Amenitsch H., Masuelli L., Peruzzi G., Mahmoudi M., Screpanti I., Zingoni A. (2019). Interplay of Protein
Corona and Immune Cells Controls Blood Residency of Liposomes. Nat. Commun..

[7] Yoneda J. S., Cardoso M. B. (2023). Nanoparticle-Induced
Conformational Changes in Protein
Corona Revealed by Circular Dichroism Spectroscopy. Nanomedicine.

[8] Srivastava I., Khan M. S., Dighe K., Alafeef M., Wang Z., Banerjee T., Ghonge T., Grove L. M., Bashir R., Pan D. (2020). On-Chip Electrical
Monitoring of Real-Time “Soft” and
“Hard” Protein Corona Formation on Carbon Nanoparticles. Small Methods.

[9] Spinozzi F., Ceccone G., Moretti P., Campanella G., Ferrero C., Combet S., Ojea-Jimenez I., Ghigna P. (2017). Structural and Thermodynamic Properties of Nanoparticle-Protein
Complexes: A Combined SAXS and SANS Study. Langmuir.

[10] Silva C. E. P., Picco A. S., Galdino F. E., de Burgos Martins de Azevedo M., Cathcarth M., Passos A. R., Cardoso M. B. (2024). Distinguishing Protein
Corona from Nanoparticle Aggregate Formation in Complex Biological
Media Using X-Ray Photon Correlation Spectroscopy. Nano Lett..

[11] Shang L., Nienhaus G. U. (2017). In Situ Characterization of Protein
Adsorption onto
Nanoparticles by Fluorescence Correlation Spectroscopy. Acc. Chem. Res..

[12] Sanchez-Cano C., Alvarez-Puebla R. A., Abendroth J. M., Beck T., Blick R., Cao Y., Caruso F., Chakraborty I., Chapman H. N., Chen C., Cohen B. E., Conceição A. L. C., Cormode D. P., Cui D., Dawson K. A., Falkenberg G., Fan C., Feliu N., Gao M., Gargioni E., Glüer C. C., Grüner F., Hassan M., Hu Y., Huang Y., Huber S., Huse N., Kang Y., Khademhosseini A., Keller T. F., Körnig C., Kotov N. A., Koziej D., Liang X. J., Liu B., Liu S., Liu Y., Liu Z., Liz-Marzán L. M., Ma X., Machicote A., Maison W., Mancuso A. P., Megahed S., Nickel B., Otto F., Palencia C., Pascarelli S., Pearson A., Peñate-Medina O., Qi B., Rädler J., Richardson J. J., Rosenhahn A., Rothkamm K., Rübhausen M., Sanyal M. K., Schaak R. E., Schlemmer H. P., Schmidt M., Schmutzler O., Schotten T., Schulz F., Sood A. K., Spiers K. M., Staufer T., Stemer D. M., Stierle A., Sun X., Tsakanova G., Weiss P. S., Weller H., Westermeier F., Xu M., Yan H., Zeng Y., Zhao Y., Zhao Y., Zhu D., Zhu Y., Parak W. J. (2021). X-Ray-Based Techniques to Study the
Nano-Bio Interface. ACS Nano.

[13] Ferreira L. F., Picco A. S., Galdino F. E., Albuquerque L. J. C., Berret J. F., Cardoso M. B. (2022). Nanoparticle-Protein Interaction:
Demystifying the Correlation between Protein Corona and Aggregation
Phenomena. ACS Appl. Mater. Interfaces.

[14] Jacques D. A., Trewhella J. (2010). Small-Angle
Scattering for Structural Biology - Expanding
the Frontier While Avoiding the Pitfalls. Protein
Sci..

[15] Vilanova O., Mittag J. J., Kelly P. M., Milani S., Dawson K. A., Rädler J. O., Franzese G. (2016). Understanding the Kinetics of Protein-Nanoparticle
Corona Formation. ACS Nano.

[16] Narayanan T. (2009). High Brilliance
Small-Angle X-Ray Scattering Applied to Soft Matter. Curr. Opin. Colloid Interface Sci..

[17] Li T., Senesi A. J., Lee B. (2016). Small Angle
X-Ray Scattering for
Nanoparticle Research. Chem. Rev..

[18] Narayanan T., Chèvremont W., Zinn T., Meneau F. (2023). Small-Angle X-Ray Scattering
in the Era of Fourth-Generation Light Sources. J. Appl. Crystallogr..

[19] Van
Vaerenbergh P., Léonardon J., Sztucki M., Boesecke P., Gorini J., Claustre L., Sever F., Morse J., Narayanan T. (2016). An Upgrade Beamline for Combined Wide, Small and Ultra
Small-Angle x-Ray Scattering at the ESRF. AIP
Conf. Proc..

[20] Cowieson N. P., Edwards-Gayle C. J. C., Inoue K., Khunti N. S., Doutch J., Williams E., Daniels S., Preece G., Krumpa N. A., Sutter J. P., Tully M. D., Terrill N. J., Rambo R. P. (2020). Beamline
B21: High-Throughput Small-Angle X-Ray Scattering at Diamond Light
Source. J. Synchrotron Radiat..

[21] Cavalcanti L. P., Torriani I. L., Plivelic T. S., Oliveira C. L. P., Kellermann G., Neuenschwander R. (2004). Two New Sealed
Sample Cells for Small Angle X-Ray Scattering
from Macromolecules in Solution and Complex Fluids Using Synchrotron
Radiation. Rev. Sci. Instrum..

[22] Pedersen, J. S. Instrumentation and Resolution Effects for Small-Angle X-Ray and Neutron Scattering. In Neutrons, X-rays, and light: Scattering methods applied to soft condensed matter; Lindner, P. , Oberdisse, J. , Eds.; Elsevier, 2025; pp 125–150. 10.1016/B978-0-443-29116-6.00025-4.

[23] Wu J., Bai X., Yan L., Baimanov D., Cong Y., Quan P., Cai R., Guan Y., Bu W., Lin B., Wang J., Yu S., Li S., Chong Y., Li Y., Hu G., Zhao Y., Chen C., Wang L. (2024). Selective regulation
of macrophage lipid metabolism via nanomaterials’ surface chemistry. Nat. Commun..

[24] Lundqvist M., Stigler J., Elia G., Lynch I., Cedervall T., Dawson K. A. (2008). Nanoparticle Size
and Surface Properties Determine
the Protein Corona with Possible Implications for Biological Impacts. Proc. Natl. Acad. Sci. U.S.A..

[25] Tenzer S., Docter D., Rosfa S., Wlodarski A., Kuharev J., Rekik A., Knauer S. K., Bantz C., Nawroth T., Bier C., Sirirattanapan J., Mann W., Treuel L., Zellner R., Maskos M., Schild H., Stauber R. H. (2011). Nanoparticle Size Is a Critical Physicochemical
Determinant of the Human Blood Plasma Corona: A Comprehensive Quantitative
Proteomic Analysis. ACS Nano.

[26] Monopoli M. P., Walczyk D., Campbell A., Elia G., Lynch I., Baldelli Bombelli F., Dawson K. A. (2011). Physical-Chemical Aspects of Protein
Corona: Relevance to in Vitro and in Vivo Biological Impacts of Nanoparticles. J. Am. Chem. Soc..

[27] Beaucage G. (1996). Small-Angle
Scattering from Polymeric Mass Fractals of Arbitrary Mass-Fractal
Dimension. J. Appl. Crystallogr..

[28] Kihara S., Aljabbari A., Be̅rziņš K., Krog L. S., Mota-Santiago P., Terry A., Kirby N., Whitten A. E., Boyd B. J. (2025). The “Gut” Corona at
the Surface of Nanoparticles Is Dependent on Exposure to Bile Salts
and Phospholipids. J. Colloid Interface Sci..

[29] Pauw B. R., Kästner C., Thünemann A. F. (2017). Nanoparticle Size Distribution Quantification:
Results of a Small-Angle X-Ray Scattering Inter-Laboratory Comparison. J. Appl. Crystallogr..

[30] Kathuria S. V., Guo L., Graceffa R., Barrea R., Nobrega R. P., Matthews C. R., Irving T. C., Bilsel O. M. (2011). Minireview:
Structural insights into
early folding events using continuous-flow time-resolved small-angle
X-ray scattering. Biopolymers.

[31] Herranz-Trillo F., Sørensen H. V., Dicko C., Pérez J., Lenton S., Foderà V., Fornell A., Skepö M., Plivelic T. S., Berntsson O., Andersson M., Magkakis K., Orädd F., Ahn B., Appio R., Da Silva J., Da Silva V., Lerato M., Terry A. E. (2024). Time-Resolved
Scattering Methods for Biological Samples at the CoSAXS Beamline,
MAX IV Laboratory. Methods Enzymol..

[32] Grant T. D., Luft J. R., Carter L. G., Matsui T., Weiss T. M., Martel A., Snell E. H. (2015). The Accurate
Assessment of Small-Angle
X-Ray Scattering Data. Acta Crystallogr., Sect.
D:Biol. Crystallogr..

[33] Jeffries C. M., Graewert M. A., Svergun D. I., Blanchet C. E. (2015). Limiting Radiation
Damage for High-Brilliance Biological Solution Scattering: Practical
Experience at the EMBL P12 Beamline PETRAIII. J. Synchrotron Radiat..

[34] Kirby N., Cowieson N., Hawley A. M., Mudie S. T., McGillivray D. J., Kusel M., Samardzic-Boban V., Ryan T. M. (2016). Improved Radiation
Dose Efficiency in Solution SAXS Using a Sheath Flow Sample Environment. Acta Crystallogr., Sect. D:Struct. Biol..

[35] Da
Vela S., Bartels K., Franke D., Soloviov D., Gräwert T., Molodenskiy D., Kolb B., Wilhelmy C., Drexel R., Meier F., Haas H., Langguth P., Graewert M. A. (2025). AF4-to-SAXS:
Expanded Characterization of Nanoparticles and Proteins at the P12
BioSAXS Beamline. J. Synchrotron Radiat..

[36] Krog L. S., Kihara S., Mota-Santiago P., Foderà V., Be̅rziņš K., Boyd B. J. (2025). Low-Frequency Raman
Spectroscopy as a New Tool for Understanding the Behaviour of Ionisable
Compounds in Dispersed Mesophases. J. Colloid
Interface Sci..

